# Development of Young Children’s Time Perception: Effect of Age and Emotional Localization

**DOI:** 10.3389/fpsyg.2021.688165

**Published:** 2021-06-08

**Authors:** Fangbing Qu, Xiaojia Shi, Aozi Zhang, Changwei Gu

**Affiliations:** College of Preschool Education, Capital Normal University, Beijing, China

**Keywords:** time perception, young children, emotional localization, facial expression, developmental course

## Abstract

Time perception is a fundamental aspect of young children’s daily lives and is affected by a number of factors. The present study aimed to investigate the precise developmental course of young children’s time perception from 3 to 5 years old and the effects of emotion localization on their time perception ability. A total of 120 children were tested using an adapted time perception task with black squares (Experiment 1) and emotional facial expressions (Experiment 2). Results suggested that children’s time perception was influenced by stimulus duration and improved gradually with increasing age. Both accuracy and reaction time were affected by the presentation sequence of emotional faces, indicating an effect of emotion localization. To summarize, young children’s time perception showed effects of age, stimulus duration, and emotion localization.

## Introduction

Time is a fundamental aspect of people’s daily lives, and the accurate perception of time may greatly aid decision-making such as judging the time left before an important meeting. Time perception is a complex process that may involve several different cognitive systems or processes such as the internal clock system, attention, and memory processes. Numerous research studies have attempted to explain the underlying mechanism of time perception. Some researchers contended that time perception depends on an internal clock inside the brain and measured this by counting the pulses emitted by a pacemaker during the presentation of a stimulus (Mauk and Buonomano, 2004; Ivry and Schlerf, 2008; Grommet et al., 2019). Other researchers argued that time perception is the outcome of an interaction between an internal clock and cognitive factors such as attention, memory, and decisional processes (Zélanti and Droit-Volet, 2011).

Despite the incompatible theories, there is a consensus in most studies on time perception that time perception ability increases with increasing age. Previous studies explained that the basic mechanisms that enable children to discriminate time duration start to be functional at an early age (Droit-Volet and Wearden, 2002; Brannon et al., 2008). Children begin to experience the change of time at a very early age and start to acquire time perception ability as early as 4 months of age (Zélanti and Droit-Volet, 2011). At the age of 3 years old, young children have similar time perception properties to human adults and animals. As they grow older, age-related effects on time perception become more significant, and children’s time sensitivity increases. Between 8 and 10 years old, children’s time sensitivity becomes close to that of adults. However, there is a limitation in previous research; most studies sampled children older than 5 years old and only a few studies have focused on children as young as 3 years old (Droit-Volet and Wearden, 2002). Gil et al. (2007) investigated age variation in temporal estimation through comparing children aged 3, 5, and 8 years old. The precise developmental course of young children’s time perception from 3 to 6 years old requires further investigation.

Recently, several researchers suggested that instead of time being interpreted independently by an internal clock system, time may be an emergent property of the neural dynamics of the brain and might not be dependent on a dedicated timing mechanism (Mauk and Buonomano, 2004; Ivry and Schlerf, 2008). Time perception may result from a complex interaction with several different cognitive factors, such as short-term memory, working memory, and selective attention (Zélanti and Droit-Volet, 2011; Droit-Volet and Zélanti, 2013). Consistent with the most influential timing model, the scalar timing model stated that time perception results from interaction between the internal clock, memory abilities, and decisional processes (Gibbon et al., 1984). According to this theory, changes in any of these processes may result in differences in time perception performance. In addition to the above factors, researchers have also found an effect of emotion modulation on time perception. Different emotional stimuli, such as facial expressions, emotion-arousing pictures, affective digitalized sounds, and emotional films, result in temporal distortions (Gil et al., 2007; Gil and Droit-Volet, 2011; Lake et al., 2016; Benau and Atchley, 2020). Different emotion valences/types have also been investigated, showing different effects on time perception. Noulhiane et al. (2007) suggested that compared with neutral sound, both positive and negative emotional sound resulted in an overestimation of time duration while the negative effect was larger. Lake et al. (2016) interpreted this negative effect on time perception as follows; negative stimuli, especially those that are threatening, have a strong relationship with human adaption, and thus easily activate one’s protecting mechanisms. Compared with neutral and happy faces, angry faces were always estimated as lasting for a longer duration in previous research. Angry faces were even judged to last for a longer duration than fearful faces (Cui, 2018). These emotional effects were explained by the fact that the perception of angry faces is arousing. This prepares the body for action and therefore accelerates the biological clock mechanism. When angry faces are perceived, the internal clock is therefore thought to generate more biological units (pulses and oscillators); with the result that time is judged to last for a longer duration.

In addition to the effect of different emotional stimuli, the localization of emotional facial expression also seems to affect time perception. According to the adopted internal clock model, the timing process includes three successive stages: clock, memory, and decision-making. Previous studies showed that the presence of emotional faces at the beginning of a stimulus presentation sequence may increase the arousal level and further accelerate the internal clock, which in turn affects the perceived time interval (Cui, 2018). However, whether this effect happens at the same stage under different types of emotional localization remains uninvestigated. Previous studies tried to test this question by setting unpredictable fear-relevant stimuli at the beginning or the end of test trials. Cui (2018) used well-designed experiments to investigate the effect of temporal localization of fearful faces on time perception. Results suggested that, compared with fearful faces presented at the beginning of a stimulus presentation sequence, there was a significant overestimation of the time duration of fearful faces presented at the end of a sequence. The fearful faces at the beginning of a sequence required more memory resources and may be more vulnerable to the effect of recency. Other studies also suggested that emotional stimuli may influence time perception by interrupting working memory processes (Fortin et al., 1993; Kensinger and Corkin, 2003).

To further investigate the influence of emotion localization on time perception, in Experiment 2 in the present study, participants were presented with neutral and angry faces in two different localization conditions (neutral-angry condition vs. angry-neutral condition) and were asked to judge which the face lasted for a longer duration. We hypothesized that the localization of angry faces may influence time perception by disrupting the process of transmission of pulses from the working memory to the reference memory.

The temporal bisection paradigm is the most frequently used in previous studies, whereby children are initially presented with stimuli with short (*S*) or long (*L*) standard durations and then presented with comparison stimuli (*t*) that either are the same as or lie between the *S* and *L* durations. The children’s task was to categorize *t* as more similar to *S* or to *L*. The psychophysical functions that have been found in young children are orderly [i.e., *p* (long) increases with the stimulus duration value], thereby revealing their ability to discriminate time. The second classical paradigm usually used in time perception studies is the temporal generalization task, in which participants compare a presented duration (longer than, shorter than, or equal to) with a standard duration stored in working memory. The third classical paradigm of time perception is the temporal reproduction task, which consists of two phases: in the first, duration is estimated and memorized and, in the second, it is reproduced after a short delay. This task also involves storing the duration to be reproduced in the first phase, followed by reproduction proper (second phase), and then comparing it with those durations previously stored in working memory (Baudouin et al., 2006).

Previous research studies have stated; however, that the above three paradigms need participants to be able to accumulate and maintain the standard/anchor durations in their working memory and then compare these with the comparison durations (Droit-Volet et al., 2011). As the stimulus duration increases, a greater load is placed on working memory (Fraisse, 1984); other cognitive abilities may lead to greater inaccuracy (Smith et al., 2002). Although there is considerable evidence that working memory abilities increase between 5 and 11 years of age (Gathercole et al., 2006), the working memory abilities of young children would still be at a relatively low level. The requirement placed on young children’s working memory by the bisection task may be beyond their actual abilities, especially for children younger than 5 years old. In studies of young children, especially those younger than 5 years old, both the temporal bisection task and temporal generalization task may be difficult for them to complete due to their limited comprehension and other cognitive abilities such as memory and judgment. Therefore, an easier experimental paradigm that can ease the burden on young children’s cognitive resources is needed.

Based on previous studies, an adapted bisection task was employed in the present study, in which the participants were sequentially presented with two stimuli with different durations (the standard duration and comparison duration) and asked to judge which of the two stimuli lasts longer. This setting eased the cognitive burden on working memory and attentional resources. In Experiment 1, an adapted bisection task was used. Children aged 3–5 years old were presented with a black square to assess the precise developmental course of time perception ability. Six different duration ranges were used: 400, 600, 800, 1,200, 1,400, and 1,600 ms as comparison durations and 1,000 ms as the standard duration. We hypothesized a steadily increasing pattern of time perception performance both in accuracy (ACC) and reaction time (RT) with increasing age. In Experiment 2, the black squares were replaced with emotional faces (angry-neutral faces or the opposite sequence, neutral-angry faces) to test whether the young children’s time perception performance was influenced by emotion localization. We expect to find that children’s time perception performance would be influenced by the sequence of angry-neutral faces, indicating an effect of emotion localization.

## Experiment 1

### Methods

#### Participants

The participants were 60 young children: 20 3-year olds (10 boys; mean age = 3.3 years, *SD* = 0.43), 20 4-year olds (10 boys; mean age = 4.7 years, *SD* = 0.46), and 20 5-year olds (10 boys; mean age = 5.2 years, *SD* = 0.40). The children were recruited from a kindergarten in Beijing, China. The Research Ethics Committee of Capital Normal University approved this study. Young children’s parents provide consent for their children’s participation in this study.

#### Materials

Young children were tested individually in a quiet room. E-Prime 2.0 software was used to present the experimental stimuli and record their response. Responses were made using the “1” and “2” keys. A 2 cm × 2 cm black square with different durations was presented in the center of the computer screen as the stimulus.

#### Experimental Procedure

**Figure 1:**
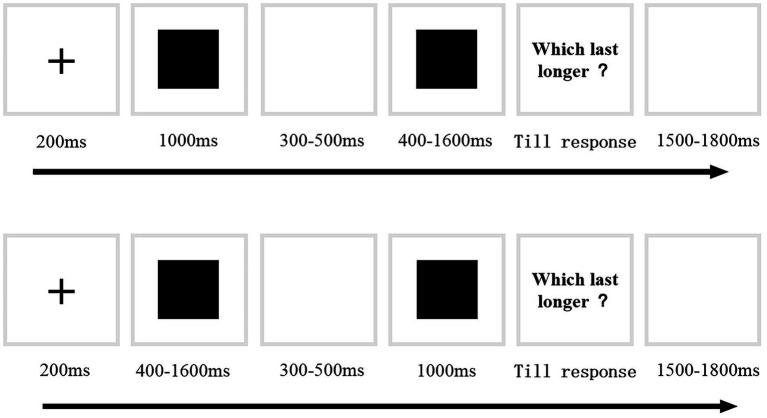
Example trial sequences used in Experiment 1.

Each participant took part in two phases: a training phase and a testing phase. In the training phase, participants were first presented with fixation for 200 ms, followed by a black square for a standard duration (1,000 ms) and then a 300–500 ms interval. After the interval, a stimulus with comparison durations (400, 1,600 ms) was presented. The presentation of the standard and comparison duration was randomized. Participants were instructed to judge who of the two squares lasted for a longer duration. A correct response would be followed by positive feedback (happy face), and an incorrect response followed by negative feedback (sad face). The training phase was terminated after a block of at least 75% correct responses. The procedure of the testing phase (see Figure 1) was the same as the training phase except that no feedback was given. The comparison duration included seven conditions: 400, 600, 800, 1,200, 1,400 and 1,600 ms (each duration was presented twice: one trial of the comparison duration followed by the standard duration and then one trial in reverse order). Each pair of comparisons was presented 10 times. Each participant completed 120 trials in total. The presentation of the trials was randomized.

### Results

A 6 × 2 × 2 repeated-measures ANOVA was employed, with stimulus duration (400, 600, 800, 1,200, 1,400 and 1,600 ms), age and sex as independent factors and ACC and RT as dependent variables. *Post hoc* simple *t*-tests were used to assess statistically significant interactions.

**Figure 2:**
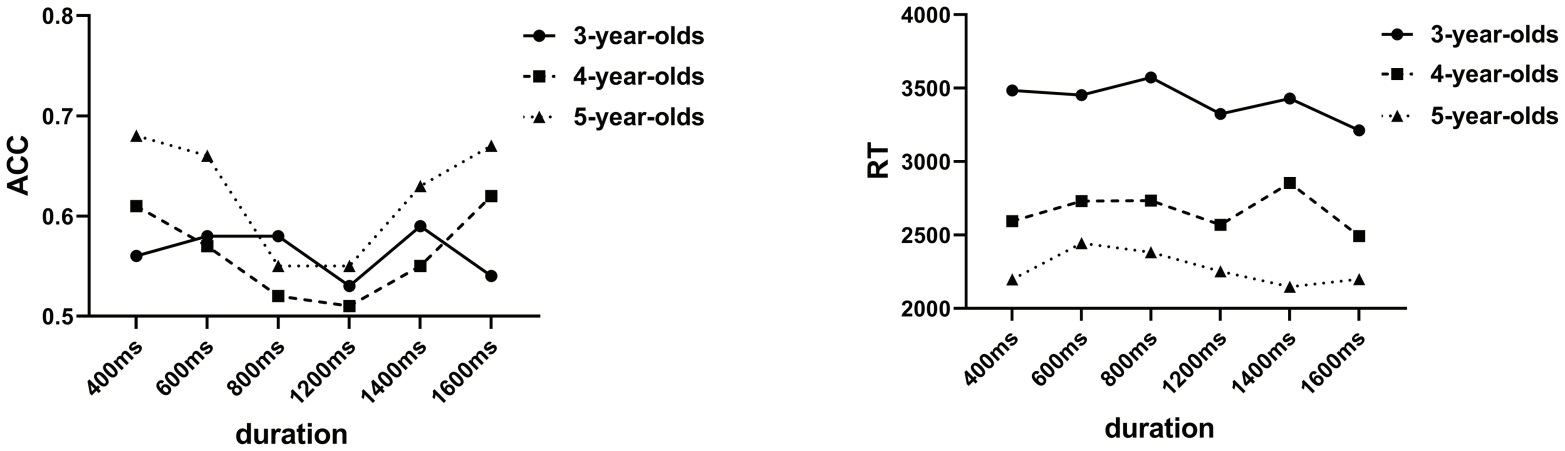
Children’s performance on time perception task (accuracy, ACC and reaction time, RT).

The ANOVA results on ACC showed significant main effects of the stimulus duration [*F* (5, 270) = 5.53, *p* < 0.001], age [*F* (2, 54) = 3.29, *p* < 0.05]. According to the *post hoc* tests (Bonferroni), the ACC under the 400, 600, and 1,600 conditions was significantly higher than that under the 800 and 1,200 conditions. The ACC under the 1,400 ms condition was significantly higher than that of 1,200 ms. The ACC of the 5-year-old group was significantly higher than that of the 3‐ and 4-year-old group. The results revealed neither a significant main effect of sex, *F* (1, 54) = 0.91, *p* = 0.35 > 0.0, nor any significant interaction among these three variables (all *p*s > 0.05; Figure 2).

Analysis of RT revealed similar results. There were significant main effects of stimulus duration [*F* (5, 270) = 2.59, *p* < 0.05] and age [*F* (2, 54) = 8.74, *p* = 0.001]. *Post hoc* analysis (Bonferroni) suggested that RT under the 1,600 ms condition was significantly shorter than the 600 and 800 ms conditions; the RT of the 1,200 ms condition was also significantly shorter than the 800 ms condition. The RT of the 4‐ and 5-year-old group was significantly shorter than that of the 3-year-old group. No significant main effect of sex [*F* (1, 54) = 1.44, *p* = 0.24] nor interaction among these factors (all *p*s > 0.05) was found.

### Discussion

The above results supported our hypothesis. First, young children’s time perception was affected by stimulus duration, with higher accuracy when the stimulus was distant from the standard stimulus (1,000 ms) in duration, as shown in the results (accuracy in the 400, 600, and 1,600 ms conditions was significantly higher than under the 800 and 1,200 ms conditions). The nearer the comparison stimulus was to the standard stimulus, the worse the accuracy was, which suggests children’s difficulty in decision-making. This result is in accordance with previous studies showed that young children tend to underestimate the duration of longer stimuli and showed higher accuracy with shorter stimuli. The reaction time results also showed the influence of duration on time perception. Judgment of stimuli with a shorter duration such as 600 and 800 ms was slower than longer durations such as 1,200 and 1,600 ms, which suggests that the subjects performed better with longer durations. This result is supported by a previous study showing that temporal discrimination was easier for short durations than for long durations (Zélanti and Droit-Volet, 2011).

Age was also a significant factor that influenced young children’s time perception. In the results, the reaction time of time perception showed a stable decreasing pattern with increasing age from 3‐ to 5-year old. This is entirely consistent with earlier bisection results obtained with older children of 5‐ and 9-year old who completed different temporal judgment tasks; the results showed that children’s sensitivity to time increases throughout children (Zélanti and Droit-Volet, 2011). The new feature of our study is that we employed a sample of younger children with a continuous age range (3–5-year old). This allowed us to show a more precise developmental course of time perception performance as a function of duration that can be discriminated from an earlier childhood perspective. The results showed that the comparison duration was more different to the standard duration; the age differences were larger with older children, who performed more accurately and faster than the younger groups. However, as the difference between the comparison and standard duration become smaller, the age differences in accuracy tended to be non-existent while the RT results still showed a stable age effect.

In Experiment 1, we tested the effect of duration and age on young children’s time perception with a non-emotional stimulus. To further investigate the impact of emotion localization on young children’s time perception, in Experiment 2, we replaced the non-emotional black square with emotional faces. We hypothesized that different emotional types may have a specific effect. Therefore, angry emotional faces were paired with neutral faces under two different localization relationships to further test the effect of emotional localization on young children’s time perception.

## Experiment 2

### Methods

#### Participants

The participants were 60 children: 20 3-year olds (10 boys; mean age = 3.2 years, *SD* = 0.40), 20 4-year olds (10 boys; mean age = 4.4 years, *SD* = 0.49), and 20 5-year olds (10 boys; mean age = 5.4 years, *SD* = 0.48). Children were recruited from a kindergarten in Beijing, China.

#### Materials

Young children were tested individually in a quiet room. E-Prime 2.0 software was used to present the experimental stimuli and record their response. Responses were made using the “1” and “2” keys. Stimuli and feedback in the training phase were same as Experiment 1. In the testing phase, stimuli consisted of 24 photographs of 12 models’ faces expressing either anger or neutrality (six males). All facial expression images from the NimStim (Tottenham et al., 2009) database were selected for the following stimuli evaluation. An additional 25 participants (18 females, *M*_age_ = 21.83 years) were recruited and asked to choose the emotion label that best describes the facial expression presented by selecting from one of the following seven emotion labels (happy, angry, sad, disgust, surprise, fear, and neutral). They also rated the intensity of the facial expression on a scale of 1–7 (from very low to very high). Stimuli selection was based on previous results (Liang et al., 2018), as well as our evaluation results. In total, 12 angry faces with high intensity and 12 neutral faces from the NimStim database were selected.

#### Experimental Procedure

**Figure 3:**
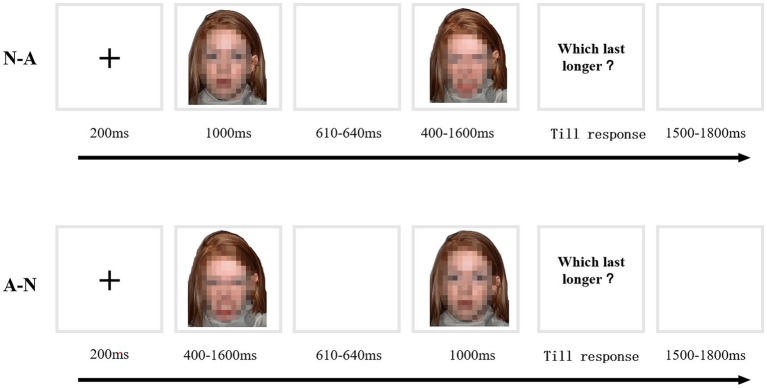
Example trial sequences: neutral-angry (N-A) condition and angry-neutral (A-N) condition.

Each participant took part in two phases: a training phase and a testing phase. The training phase was the same as Experiment 1. In the testing phase, the black square was replaced by emotional faces (see Figure 3). A neutral face was used as the standard stimulus and angry faces were used as comparison stimuli. Two emotion localization conditions were formed: a neutral-anger condition (N-A) and an angry-neutral (A-N) condition. Each pair of comparisons was presented 10 times. Each participant completed 120 trials in total. The presentation of the two conditions was randomized.

### Results

#### ACC Results

A 6 × 2 × 2 repeated-measures ANOVA was employed, with stimulus duration (400, 600, 800, 1,200, 1,400 and 1,600), age, sex, and temporal localization of emotion as independent factors and ACC and RT as dependent variables. *Post hoc* simple *t*-tests were used to assess statistically significant interactions.

The ANOVA results on ACC showed significant main effects of the stimulus duration [*F* (5, 270) = 4.49, *p* < 0.001, *η*^2^*_p_* = 0.04], age [*F* (2, 54) = 6.98, *p* < 0.001, *η*^2^*_p_* = 0.11], and temporal localization of emotion [*F* (2, 54) = 18.1, *p* = 0.001, *η*^2^*_p_* = 0.13]. According to the *post hoc* tests (Bonferroni), the ACC under the 400 ms condition was significantly higher than that under the 600, 800, 1,200, and 1,400 ms conditions, while it showed no difference under the 1,600 ms condition. The ACC less than 1,600 ms was significantly higher than that of 1,200 ms.

**Figure 4:**
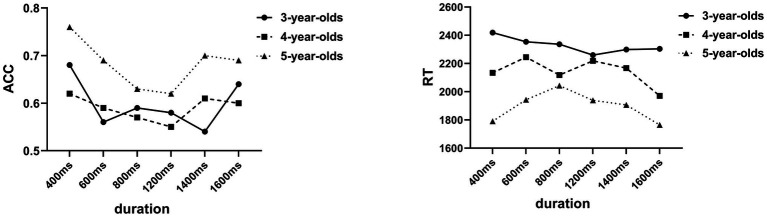
ACC and RT under different durations and age.

**Figure 5:**
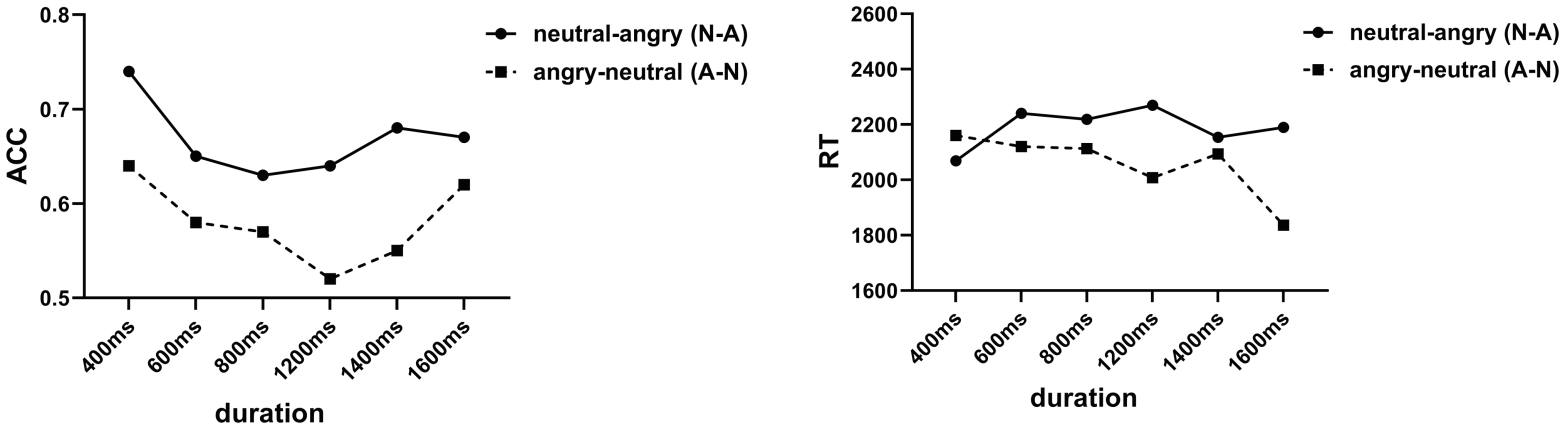
ACC and RT under different durations and emotional localization.

The ACC of the 5-year-old group was significantly better than the 3‐ and 4-year-old groups. The ACC under the N-A condition was significantly higher than that of the A-N condition. An interaction effect was found between age and temporal localization of emotion [*F* (2, 54) = 3.84, *p* < 0.05, *η*^2^*_p_* = 0.07]. A simple effects analysis showed that the ACC of the 5‐ and 4-year group under the N-A condition was significantly higher than that of the A-N condition. The ACC of the 5-year-old group was significantly higher than that of the 3‐ and 4-year-old groups under the N-A condition. No main effect of sex, *F* (1, 54) = 0.91, *p* = 0.35 > 0.05, nor any significant interaction among the other variables were found (all *p*s > 0.05; Figures 4, 5).

#### RT Results

Analysis of the RT results revealed similar findings. A main effect of age (*F* = 4.08, *p* = 0.02 < 0.05, *η*^2^*_p_* = 0.07) and an interaction between duration and temporal localization of emotion was found (*F* = 2.67, *p* = 0.02 < 0.05, *η*^2^*_p_* = 0.02). No significant main effect of sex (*F* = 0.14, *p* = 0.72, *η*^2^*_p_* = 0.01) and temporal localization of emotion (*F* = 1.19, *p* = 0.28, *η*^2^*_p_* = 0.02) nor interaction among these factors (all *p*s > 0.05) was found. *Post hoc* analysis (Bonferroni) suggested that the RT of the 5-year-old group was significantly shorter than that of the 3-year-old group. Simple effects analysis showed that the RT of 1,600 ms was significantly shorter than that of 400 ms under the A-N condition.

### Discussion

The results obtained in Experiment 2 provide convergent evidence to the effect of duration and age on time perception found in Experiment 1. The closer the stimulus duration to the standard duration the lower the accuracy. Analysis of the age effect of ACC and RT showed a similar pattern to Experiment 1: with increasing age from 3 to 5 years old, the ACC increased and RT decreased. Emotion has been shown to influence the perception of time (Gil et al., 2007; Gil and Droit-Volet, 2011; Lake et al., 2016). However, no experiment has studied whether the presentation sequence of emotional faces would affect one’s time perception. Furthermore, Experiment 2 showed an effect of emotion localization on young children’s time perception, in which the neutral-angry condition achieved significantly better accuracy with no significant difference in reaction time. In one recent study, researchers investigated the effect of expectancy on time perception by manipulating the sequence of fearful faces, and results suggested in predictable condition, the emotion effect was disappeared (Cui, 2018). In present experiment, compared with the neutral-anger condition, the anger-neutral condition can be seen as a predictable condition and thus may have no effect on the time perception. While under the neutral-anger condition, the participants may exert more resources on the processing of relatively unpredictable angry faces and result in higher accuracy.

## General Discussion

Stimulus duration whether a non-emotional black square or emotional facial expression significantly influenced the young children’s time perception accuracy. The closer the duration between the comparison and standard stimulus, the lower the accuracy. In addition, older subjects showed better performance on the judgment of both non-emotional black squares and the emotional facial expression, which suggests the age effect on time perception was stable regardless of stimulus type. We also observed an emotion localization effect on time perception. Compared with emotional faces presented before the neutral faces condition, the emotional faces presented after the neutral faces condition showed better accuracy, indicating that the time discrimination task was influenced by the localization of the emotional faces.

### Age-Related Effect

The present study clearly showed that time perception ability increases throughout the early years of childhood both in terms of accuracy and reaction time of judgment. This is entirely consistent with earlier studies conducted with children. The originality of our study lies in the participants tested constituting a continuous age range (3–5-year old), while most previous studies employed young participants with a discontinuous age range. This allowed us to further investigate the precise developmental course of time perception ability as a function of duration. When the difference between comparison and standard duration was bigger, such as 400/600/1400/1600 vs. 1,000 ms, all three age groups showed higher accuracy and faster reaction time. A clear age difference was also found with older children who performed better. The accuracy and reaction time were impacted when the difference was narrowed, as shown in the 800/1200 ms vs. 1,000 ms condition. However, the 5-year-old group still showed better performance compared with the 3‐ and 4-year-old groups. This result is supported by previous results, suggesting that the ability to discriminate time duration increases with improvement in several important cognitive abilities in children, such as working memory, and executive functions, such as selective attention and inhibitory capacity (Zélanti and Droit-Volet, 2011).

### Emotional Localization Effect

Most previous studies focused on the effect of different emotion types on time perception, while neglecting the possible effect of emotion sequence. Our study showed that the temporal localization of emotional faces also had an effect on the performance of time perception, with the results suggesting that young children scored higher in the neutral-angry condition than in the angry-neutral condition. As suggested in previous studies, we may assume that the emotion localization effect found in Experiment 2 was due to the automatic activation of the nervous system by the angry faces. Since the internal clock system depends on the dynamic functioning of the brain, when the angry faces are presented, its rate increases and produces more time units for the processing of the stimulus. Thus, the angry faces presented last in the stimulus presentation sequence were processed for a longer duration and judged better than when angry faces were presented first in the angry-neutral condition (Droit-Volet et al., 2011). This effect is also in accordance with the theory of attention bias. Previous studies have reported that the negative valence of target stimuli modulate one’s attention, such that angry faces are almost automatically detected in the visual search task (Hansen and Hansen, 1988; Williams et al., 1996; Bradley et al., 1998; Eastwood et al., 2001; Miyazawa and Iwasaki, 2010).

Studies on the effect of angry faces on time perception also showed that angry faces induce an overestimation of time perception in both adults and children (Gil and Droit-Volet, 2011). Furthermore, previous studies showed that an expectation of negative stimuli modulates time perception, whereby the duration of the stimulus was judged to be longer as arousal level increased in response to an unexpected threatening stimulus (Droit-Volet et al., 2010; Cui et al., 2018). In the neutral-angry condition, the angry face was presented last, which may induce the subjects to form an expectation of unpredictable negative stimuli and so attract more attention and processing time, thus achieving better accuracy. Under the angry-neutral condition, the angry face was presented first and thus attracted less attention and shorter processing time, which was reflected in relatively lower accuracy.

### Duration Effect

The results also showed that young children’s time perception was influenced by the duration of the stimulus. Young children performed better when the difference between the comparison and standard duration was larger, as shown under the 400, 600, and 1,600 comparison conditions. The reaction time results also showed the same pattern with subjects performing faster under the larger difference condition. These results are in line with the mathematics of internal clock models, which suggested that a clock rate-based mechanism needs a multiplicative effect in which the duration value influences the temporal judgment (Droit-Volet et al., 2011). In accordance with the present results, previous studies have demonstrated that different duration intervals may have different demands on attention and cause an effect of duration on time perception. A duration boundary may exist that involves distinct cognitive processes, which lies at approximately 1 s (Lewis and Miall, 2006, 2009; Ivry and Schlerf, 2008). Short duration was seen to involve less attentional resources and to be more automatic, while longer duration may recruit high-level cognitive capacities (Lewis and Miall, 2006; Ivry and Schlerf, 2008; Meck et al., 2008).

This study had some limitations that should be addressed. First, in this paper, a new adapted time perception paradigm was used to ease the cognitive demand on young children’s ability. However, previous studies have found that the temporal task used may influence the effect of emotion on time perception (Gil and Droit-Volet, 2011). Further comparison analysis should be conducted to investigate the effect of the paradigm used that may confound young children’s time perception performance. Second, compared with using Weber Ratio (WR) and point of subject equality (PSE) as indicators of time perception ability in previous studies, we used ACC and RT in the present study. In future studies, the effect of emotion localization could be further studied with traditional indicators such as WR and PSE to see how the influence was achieved.

## Conclusion

To summarize, our experiments, which used an adopted bisection task with different comparison durations and stimuli (non-emotional black square and emotional faces), revealed a significant age-related increase in accuracy and a decrease in reaction time and indicated that young children’s time perception ability increases gradually and significantly from 3‐ to 5-year old. Analyses on the effect of emotion localization revealed that the effect of emotion on time perception was influenced by the temporal sequence of angry face before neutral face.

## Data Availability Statement

The raw data supporting the conclusions of this article will be made available by the authors, without undue reservation.

## Ethics Statement

The studies involving human participants were reviewed and approved by the Ethics Committee of the College of Preschool Education, Capital Normal University. Written informed consent to participate in this study was provided by the participants’ legal guardian/next of kin.

## Author Contributions

FQ and XS contributed to designing the experiments and analyzing the data. XS and AZ contributed to collecting the data. FQ and CG contributed to writing the manuscript. All authors contributed to the article and approved the submitted version.

### Conflict of Interest

The authors declare that the research was conducted in the absence of any commercial or financial relationships that could be construed as a potential conflict of interest.
